# An elucidation of over a century old enigma in genetics—Heterosis

**DOI:** 10.1371/journal.pbio.3000215

**Published:** 2019-04-24

**Authors:** Diddahally R. Govindaraju

**Affiliations:** 1 Museum of Comparative Zoology, Harvard University, Cambridge, Massachusetts, United States of America; 2 The Institute for Aging Research, Albert Einstein College of Medicine, Bronx, New York, United States of America

## Abstract

Recognition and exploitation of hybrid vigor or heterosis among individual crosses of plants and animals has a long and distinguished history. Its manifestation is influenced by a combination of genetic, epigenetic, phenotypic, and environmental factors. Although heterosis is known to be governed by both dominant and epistatic gene action, its expression is greatly influenced by nonlinear interaction among epigenetic and phenotypic (phenomic) components. The magnitude of heterosis is generally inferred post hoc by the phenotypic performance of hybrids among laboriously made individual crosses. The expression of dominance, however, is nonlinear at the cellular level and obeys the principles underlying metabolic flux. Then, is it possible to exploit these relationships to predict heterosis? Vasseur and colleagues have indeed demonstrated the feasibility of such an approach in a series of experiments taking integrated biochemical and computational approaches, as well as testing these results on large samples of model organisms. The results offer promise toward phenomic prediction of heterosis across a wide array of organisms.

## Abstract 2

A recent study applies allometric models to test Sewall Wright’s 85 year-old conjecture on the expression of dominance among traits that contribute to productivity and Darwinian fitness in Arabidopsis thaliana. This Primer explores the wide-ranging implications for agriculture and human health.

“… cross-fertilisation is generally beneficial, and self-fertilisation injurious. This is shown by the difference in height, weight, constitutional vigour, and fertility of the offspring from crossed and self-fertilised flowers, and in the number of seeds produced by the parent-plants…. we may infer that with mankind the marriages of nearly related persons, some of whose parents and ancestors had lived under very different conditions, would be much less injurious than that of persons who had always lived in the same place and followed the same habits of life.”[1]

“… so far, no generally applicable law governing the formation and development of hybrids has been successfully formulated can hardly be wondered at by anyone who is acquainted with the extent of the task, and can appreciate the difficulties with which experiments of this class have to contend.”[2]

## Hybrids and hybrid vigor in evolution and genetics

The question of why certain progeny of crosses or hybrids between unrelated individual plants and animals show greater vigor, productivity, disease resistance, viability and fertility, or Darwinian fitness as opposed to the progeny of close relatives has fascinated humans for millennia [3,4]. As quoted above, both Darwin and Mendel explored this widespread biological phenomenon and its inheritance among interindividual crosses from different perspectives. Therefore, it may not be an exaggeration to state that inquiries into the causes and consequences of the origin and inheritance of variation, as well as the observed superiority or inferiority of hybrids, lie at the foundation of both evolution and genetics, and of course plant and animal breeding. Whereas Darwin recorded the ubiquity of “greater innate constitutional vigour” of hybrids for height, weight, and fertility among diverse plant taxa [1], Mendel investigated mechanisms underlying the formation of primarily 2 classes of individuals, namely, recessives and dominants, among the segregating progeny of hybrids derived from individual crosses. Following the rediscovery of Mendelian laws, subsequent investigators, notably Bateson and Garrod, discovered that the 2 classes (3:1) described by Mendel could be represented by three different categories of individuals in the segregating populations (i.e., AA homozygote dominants and Aa heterozygotes and aa recessive genotypes; 1:2:1). Individuals in each of these classes could exhibit distinct morphological and physiological (phenotypic) properties. Important among them is that the phenotypes of heterozygotes were either intermediate or approached the dominant genotypes. Many others, however, found that the expectation that crosses between two parents as intermediate was frequently violated. Instead, individuals from heterozygote classes were often superior to their parents in size, growth rate, fertility, drought, and disease resistance, etc., commonly referred to as hybrid vigor. Shull [5], in particular, demonstrated a dramatic increase in the yields of hybrids between inbred lines of corn relative to their parental lines. This success, repeated in many crops since, has been hailed as “one of genetics’ greatest triumphs” [6].

## Genetic explanations of hybrid vigor

**Fig 1:**
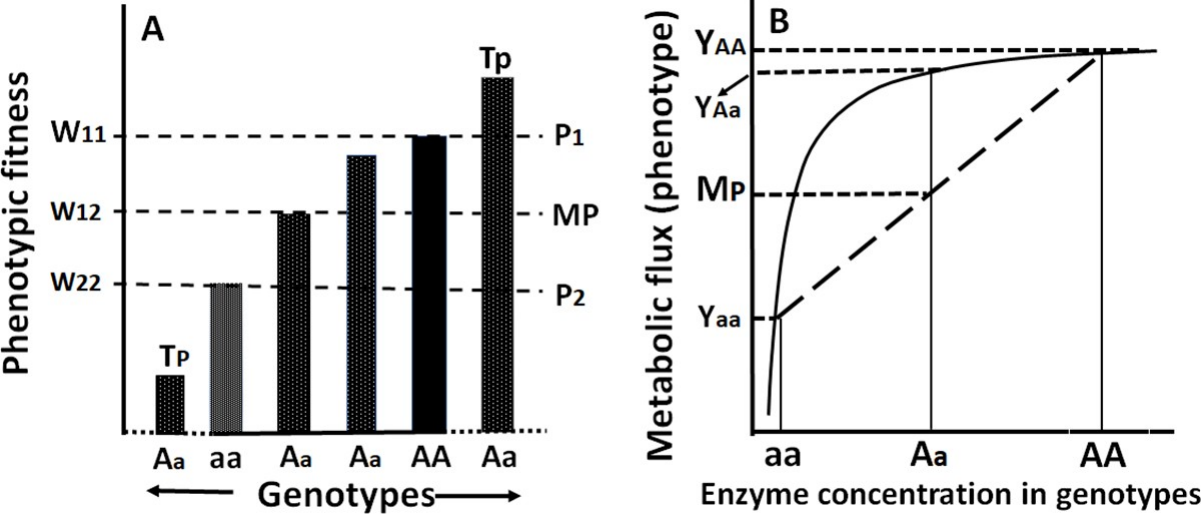
Phenotypic expression of dominance in different genotypes, their fitness and enzyme flux. (A) Major classes of phenotypic diversity and their fitness in interindividual crosses of homozygote recessive (aa; P2, worst parent) and dominant (AA; P1, best parent) genotypes. With monogenic inheritance, phenotypic expression of heterozygotes (Aa) could deviate from (1) MP approaching dominants (best parent) and (2) inferior or superior (Tps) to both recessive and dominant genotypes (worst and best parents, respectively). (B) Wright’s view of the phenotypic manifestation of dominance of genotypes as a function of enzyme flux. Note the flux of heterozygotes exceeds midparent values (modified from [26]). MP, midparent; Tp, transgressive phenotype.

What are the biological bases of hybrid vigor? A question that has nagged geneticists for over a century. Because hybrid vigor is the manifest property of heterozygotes, at least 2 explanations are popular. First, according to Mendel’s views, both heterozygote and dominant genotypes are treated equally because the expression of the recessive allele is suppressed by the dominant allele; therefore, the expression of heterozygotes can hardly be distinguished from the (dominant) wild types. Second, the expression of heterozygotes could fall midway between recessives and dominants (midparent) or occasionally even exceed the best parent (overdominant). In other words, according to dominance hypothesis, the harmful effects of recessive alleles carried by parental gametes are suppressed, retaining only the effects of favorable dominant alleles. The overdominance hypothesis, on the other hand, attributes heterosis to superior fitness of heterozygous genotypes over homozygous wild type (best parent) [7,8]. Therefore, dominance, overdominance, and underdominance (hybrid inferiority) represent a range of phenomic (extra-genomic aspects [9]) expression for one or many traits among progeny of individual crosses (Fig 1A).

## Fisher’s and Wright’s views of dominance

Among many ideas put forward to explain the phenotypic superiority of hybrids or heterosis, genetic and physiological (epigenetic) theories offered, respectively, by Fisher [10] and Wright [11], are the most discussed [12]. Fisher argued that dominance of an allele may be attributed to evolutionary processes and heterozygotes are naturally intermediate (Fig 1A) between the recessive and the wild-type (dominant) genotypes, but the frequently observed superior phenotypic expression of heterozygotes may be attributed to the accumulation and maintenance of modifiers in an additive manner. Sewall Wright [13], on the other hand, argued that “…dominance is a phenomenon of physiology of development to be associated with the various types of epistatic relationships among factors rather than with the more fundamental genetic principles,” as advocated by Fisher. This stance disrupted even their personal relationships [14]. Controversies aside, Wright explored this concept further in one of his great, yet moderately known papers (it has received only 319 citations in 85 years!), titled, “Physiological and evolutionary theories of dominance” [13]. This paper at once illuminates many ideas that are central to evolutionary biology and genetics: (a) illustrates the role of enzymes, as primary products of genes among cell differentiation and developmental pathways in relation to environment; (b) views “development is (as) an epigenetic process”; (c) suggest that genes do not show “one-to-one relation to morphological characters” and instead interaction and pleiotropy are ubiquitous; (d) introduced (from the perspective of the present discussion), nascent concepts emerging from enzyme kinetics to explain the relative levels of phenotypic expression of recessive and dominant homozygotes as well as heterozygotes; (e) links gene action to cascading levels of biological organization; (f) demonstrates an intimate relationship among genotype diversity, enzyme kinetics, and their phenotypic expression (flux), which assumes curvilinear pattern and ultimately plateaus out; and finally, (g) laid a foundation toward developing causal, systems and network analysis in genetics and biology. Sewall Wright’s views and theories on dominance published 85 years ago, in my opinion, paved the way for further exploration and growth of epigenetics, gene-enzyme concepts, metabolic flux, understanding heterosis, genotype–phenotype (G–P) mapping, and evolutionary genetics, to name a few.

## Relationships among genotypes, metabolic flux, phenotypic expression, and fitness

In brief, Wright as a biologist, was quick to grasp the scope of new discoveries emerging from biochemistry and extend them to explain the phenomenon of dominance as well as the relationships among genes, enzymes, intermediate developmental processes, and, ultimately, the phenotype. He reasoned that enzymes as primary gene products act on substrates and their products generally accumulate in a nonlinear fashion and deviate from strict additivity. The rate of production of enzyme products in a given pathway or fluxes are, in fact, phenotypes in the epigenetic (physiological) and phenotypic spaces (“phenomic” [9]). The flux produced by recessive, heterozygote, and dominant genotypes (Fig 1B) could also be modulated by environmental factors [15,16]. Wright’s idea was further elaborated by Kacser and Burns [17,18] for an ensemble of enzymes among systems of biochemical pathways and showed that activity of any one enzyme in the system has only a minor influence on the performance of the overall system or the phenotype. This theory, what has since come to be known as “metabolic control theory” (MCT), shows synergistic and antagonistic and emergent properties of enzymes in a polygenic system that influences all quantitative traits [19]. Because enzyme-substrate complexes show curvilinear relationships in such a system, the rate of enzyme reaction and amount of product may be predictable in accordance with the properties of enzyme kinetics, which obey allometric scaling rules at the epigenetic and phenotypic (phenomic) levels of organization [20]. Accordingly, a large body of theoretical and experimental evidence lends support to the predictable features embedded in the curvilinear aspects of MCT [19]. Deviation from additive effects is also a common feature of phenotypes and their components because of their functional interactions and integration at all levels of an organism during growth and development [21–23].

## Creation of enzyme hybrids and studying their heterotic properties in test tubes

Because metabolic fluxes provide a basis for physiological epistasis and expression of heterosis among phenomic components [24], could heterosis be simulated in vitro and predicted subsequently among individual crosses of plants? In fact, Fievet and colleagues [25] provided a convincing answer to a set of these questions. They artificially synthesized 61 enzyme hybrids involving 36 enzyme parents in test tubes, all of which showed either superior (positive) or inferior (negative) heterosis, relative to the midparent values, as expected among the hybrid progeny of individual crosses. They evaluated the consistency of these results using computer simulations and tested further on a part of the glycolytic pathway involving yeast crosses. All of these results consistently validated Wright’s as well as Kacser and Burns’ conjectures on dominance.

## Nonlinear allometric properties of parental phenotypes may serve as predictive indices of heterosis in their crosses

As a logical extension to Fievet and colleagues’ study, Vasseur and colleagues [26] set out to further verify the nonlinear relationships and integrity of inheritance of 4 phenotypic traits among 451 hybrids derived from crossing *Arabidopsis thaliana* ecotypes drawn from a wide geographical area around the world. They measured the expression of heterosis among hybrids as a deviation from midparent on 4 traits—growth rate, age at reproduction, biomass, and fruit number. These traits represent, respectively, viability and reproductive components of Darwinian fitness. The authors also reported that the nonlinear allometric relationship between growth rate and its components of parents and their progeny explained nearly 75% variance for heterosis, but geographic distance accounted for only 7% for the same traits. Their results suggest that crosses between geographically widely (and therefore genetically) isolated lines might suffer from Dobzhansky-Muller incompatibilities [27]. The observed heterosis between crosses reflects the allometric and multiplicative property of gene products in the epigenetic and phenotype spaces [9,28]. There is large body of theoretical and empirical work on the pervasive nonlinear allometry from molecules to ecosystmes, called MST [29–31]. Despite these conceptual advances, there is still a need for wide-ranging applications of MST in the analysis of evolutionary genetic basis of quantitative traits. Vasseur and coworkers have innovativly integrated the idea of metabolic scaling to test Wright’s prescient theory of dominance associated with productivity and also Darwininan fitness of individuals. Additionally, their work provides a convincing biochemical explanation underlying offspring–parent phenotypic resemblance, which is central to quantitative genetics [32,33].

Contrary to claims made by many genome-wide association studies (GWAS), this study suggests that additive properties of gene action may not fully capture the nonlinear nature of gene action and thus insufficient to predict phenomic manifestation of metric traits in relation to environmental variation. In polygenic systems, both *cis* and *trans* interactions (epistasis) of genes, gene products (enzymes), as well as interaction among correlated traits, influence the magnitude of heterosis in the entire phenotype and its component traits. Perhaps, in the future, the performance of hybrids could be evaluated in vitro for their superior specific combining abilities on the bases of a panel of parental phenomic properties bypassing the laborious field testing of hybrids developed from complex breeding designs [34]. Additionally, because it is well known that inbreeding leads to hybrid inferiority and increased susceptibilities to pests and diseases, for conservation of endangered species, parents could be evaluated a priori such that the progeny would show greater homeostatic properties [35]. Besides exploitation of heterosis, a knowledge of the causes and consequences of inter- and intraspecific hybridization in natural populations of plants and animals [36] would be helpful in wide-ranging fields of production agriculture and evolutionary biology: maintenance of varietal and/or stock purity, commercial seed production, designing seed zones, understanding hybrid zones, and speciation.

In humans, nearly 10% of the global populations are known to practice consaguineous marriages, which could be as high as 50% in some countries. Consanguinity is known to increase the incidence of many recessive and complex disorders [37]. Recent trends in urbanization point toward reduced levels of consanguinity, which often leads to elevated levels of heterozygosity and purported health benefits [38]. With proper ethical and legal consent, increasing the levels of heterozygosty among children of prospective parents could serve as a useful tool toward improving population health in the arsenal of genetic counseling. For instance, a biochemical view of gene action would be useful in understanding the penetrance and expressivity of alleles [39] in nearly 7,000 Mendelian disorders and perhaps for devising medical interventions [40]. Heterozygosity measured using protein or DNA markers also show a general curvilinear relationship with quantitative traits, but the magnitude of relationships changed in relation to environments as predicted by the amplitude for expression of heterosis in Wright’s model [16,41,42]. Similarly, methylation, an epigenetic marker, shows both linear and nonlinear as well as a predictable relationship with human longevity [43], which is a complex quantitative trait. Biomass, like longevity, studied by Vasseur and colleagues is equally complex because it “integrates the number of metabolically active units (cell, mitochondria)” and also “determines physiological (epigenetic) fluxes, biomass partitioning, growth rate and metabolic activity at the organismal level.” Phenotypes are products of sequential and hierarchical differentiation and conjugated multilevel system of traits, which show developmental and functional integration, interaction, and modification [23], and selection could act on any one of the components or the entire organism. The integration and influence of phenomic traits cut across all levels of the genotype–epigenetic–phenotype (G–E–P) space [9,28]; therefore, in principle, they are predictable across all levels of biological diversity [21] as well. These basic principles also suggest that heterosis could influence any of these components singly or the entire correlated system, as well as confer both flexibility and robustness in the face environmental uncertainties in diverse living systems [41,42]. This work not only validates Wright’s prescient ideas on the origins of hybrid vigor but also attempts to unveil the mystery surrounding the expression of hybrid vigor. Despite countless numbers of studies on the manifestation of heterosis in diverse organisms gathered over decades, simple biological principles could remain elusive. Insights from the original masters still matter in order to make conceptual leaps in our understanding of several important and ubiquitous biological phenomenon such as heterosis. The present study offers novel and integrated approaches for further exploration and exploitation of heterosis in ecology and evolutionary biology, plant and animal breeding, as well as in the amelioration of human health.

## Abbreviations

G–E–P: genotype–epigenetic–phenotype
G–P: genotype–phenotype
GWAS: genome-wide association studies
MCT: metabolic control theory
